# Fair Processes for Priority Setting: Putting Theory into Practice: Comment on "Expanded HTA: Enhancing Fairness and Legitimacy"

**DOI:** 10.15171/ijhpm.2016.85

**Published:** 2016-07-03

**Authors:** Maarten P. Jansen, Jan-Kees Helderman, Bert Boer, Rob Baltussen

**Affiliations:** ^1^Radboud Institute for Health Sciences, Radboud University Medical Center, Nijmegen, The Netherlands.; ^2^Institute for Management Research, Radboud University, Nijmegen, The Netherlands.; ^3^Institute of Health Policy and Management, Erasmus University Rotterdam, Rotterdam, The Netherlands.

**Keywords:** Priority Setting, Healthcare Technology Assessment (HTA), Values, HTA as Learning, Fair Processes, Evidence-Informed Deliberative Processes

## Abstract

Embedding health technology assessment (HTA) in a fair process has great potential to capture societal values relevant to public reimbursement decisions on health technologies. However, the development of such processes for priority setting has largely been theoretical. In this paper, we provide further practical lead ways on how these processes can be implemented. We first present the misconception about the relation between facts and values that is since long misleading the conduct of HTA and underlies the current assessment-appraisal split. We then argue that HTA should instead be explicitly organized as an ongoing evidence-informed deliberative process, that facilitates learning among stakeholders. This has important consequences for whose values to consider, how to deal with vested interests, how to consider all values in the decision-making process, and how to communicate decisions. This is in stark contrast to how HTA processes are implemented now. It is time to set the stage for HTA as learning.

Daniels and colleagues have recently called for an expansion of the practice of health technology assessment (HTA), in order to capture societal values relevant to public reimbursement decisions of health technologies.^1^ We agree with their suggestion that embedding HTA in a fair process has great potential in achieving this, in the sense that this allows for the inclusion of stakeholder values. We see this an important development in the revision of theory and tools for priority setting – by moving away from rather technocratic approaches that merely aim to maximize health gains, to, instead, interpreting priority setting as a value laden political process, in which the use of evidence-informed deliberative processes contributes to setting legitimate priorities in health.^1-3^

Yet, as we see it, the development of such processes for priority setting has largely been theoretical. A companion article stresses the need for development and implementation of evidence-informed deliberative processes.^3^ In this paper, we propose further practical lead ways on how these processes can be implemented. We argue that this requires the inclusion of stakeholder’s values and expertise, and this can only be realized if HTA is explicitly organized as an ongoing learning process among stakeholders – to acquire shared knowledge on what is valuable about a specific health technology^4^ and help negotiate between vested interests of stakeholders. This is in stark contrast to how HTA processes are implemented now. We present this misfit, and then proceed by providing practical lead ways on how HTA as learning can be implemented. We argue that these processes of learning about the facts and values of priority setting need to be proactively coordinated.^5,6^ This is how we believe that HTA can best contribute to setting fair and legitimate priorities in health.

## The Misconception About Facts and Values

It is widely acknowledged that present HTA processes fall short to provide decision-makers with a comprehensive set of information to base their decisions on.^1,7-9^ Especially, HTA processes do not provide decision-makers with proper guidance on how they should judge ethical issues. With others, we argue that an important reason is the conduct of HTA in two separate phases: (*i*) the generation of evidence itself, or the assessment phase and (*ii*) the interpretation of the evidence collected, or the appraisal phase.^10^ The mistaken underlying belief is that the assessment phase is a value-free kind of scientific research – which produces objective data for the appraisal phase, during which then values are brought to bear on the available evidence. This distinction is utterly misconceived, since it assumes we can collect facts in a value-free and completely objective way.^10^ In reality, value-based choices are already made at the point of choosing what evidence needs to be collected.

HTA agencies that have institutionalized this separation often choose to use a standard set of criteria during the assessment phase, pushing the consideration of other criteria into the appraisal phase. The consequence is that the evaluation of further concerns – which are not automatically covered by the standard criteria – becomes a post-assessment assessment exercise. Evidence on these aspects will not be collected or it will not be in time for use in the decision-making process.

A demonstration of the misconception about the relation between facts and values is the prominent use of cost-effectiveness analysis (CEA) as a method of evaluation in HTA. There is little explicit recognition that the choice to conduct a CEA is already dependent on valuing health maximization in the first place and that it does not adequately reflect all relevant societal values. Worse yet, many of the ‘objective’ criteria that HTA relies on in its assessment phase, depend to a large extent on the way they work out in operational practice.^11^

## Organizing Health Technology Assessment as a Learning Process

Organizing ‘HTA as learning’^4,10-13^ instead requires that well-organized deliberative processes and procedures are established that induce and help stakeholders spell-out what they find relevant values at the very start of the decision-making process. Also, it is key that stakeholders reflect upon evidence whenever it is, or becomes, available – they need to ask themselves what the evidence means to them in their current practices and what new questions are relevant to answer. This includes a deliberation on initial constraints and conditions that may hinder the approval of the technology, and whether and how these can be overcome so that a positive or provisional decision comes within the realm of politically legitimated conditions.^6,14^ This appraisal among all public and private stakeholders must continue throughout the process, until the end, when politically authorized decision-makers have to reach a final (or provisional) decision. As such, the process must not be organized as a two-phase process – separating assessment and appraisal. Instead, it should be organized as a continuous interactive and dialectical exploration of what is valuable (or what gives stakeholders reason for concern) about the health technology at stake.

The underlying assumption is that stakeholders’ understanding of the technology, the disease or its further context may change, or evolve, when stakeholders participate in a learning process among stakeholders. This requires from stakeholders that they are both able and willing to learn, and experimental and anecdotal evidence supports this.^15-18^ Also, it is assumed that such new understanding is an improvement, compared to the initial understanding of stakeholders, and that decision-making based on this new understanding is better able to provide both stakeholders and citizens outside the process with well-justified reasons to confer legitimacy to the decision-making process and its final decision.

## Whose Values Are Important?

In order to identify all relevant values, knowledge and questions, all stakeholders who are somehow affected by the to-be-made decision should be able to participate in the process – either indirectly represented via appraisal committees, interest groups, organizations, or directly as patients, healthcare workers or citizens (taxpayers). If not all stakeholders are involved, potentially valuable insights are easily missed out on – hindering the learning process.

In particular, there is a strong normative demand to provide people who are adversely affected by priority setting decisions with well-justified reasons for conferring legitimacy to the decision.^19^ This implies that HTA processes that aim to optimize priority setting decisions – with the aim of increasing the fairness and legitimacy of these processes – should be organized in such a way that special attention is given to consult those who are adversely affected by decisions each time a tentative decision is reached. This specific (conflict) interaction can at the same time be an important driver for learning and produce relevant questions for further assessment.

## Are Public Values the Sum of Private Values?

Stakeholders often have strong vested interests and it should not come as a surprise that they initially push in favour of these interests. This has two consequences. Firstly, in practice, unregulated discussions and unorganized deliberative processes easily end up in unending disputes. Especially so, when stakeholders are only involved at the end of the decision-making process, during the appraisal phase –giving stakeholders the impression there are only limited opportunities left to have an impact on decision-making and offering them little time to learn from other perspectives and arguments. This again indicates the need to regulate deliberative processes, and involve all stakeholders right from the start. Such regulation should clarify how stakeholders can contribute to the process, rules of argumentation, weighing, trading-off arguments and should make explicit how the final decision is to be taken.

Second, stakeholder consultation in the presence of vested interests likely captures private values, but it is less able to capture specific public interests that we have good reasons to care about, such as safeguarding equal access to good quality healthcare, efficiency and cost containment. Such public interests are not per se endorsed or defended as important by (private) stakeholders. Therefore, if we agree on the importance of specific public interests, that are the product of (inter)national learning about what should count as legitimate public interests, their consideration and inclusion into the learning process must be organized – in a legitimate way. This may translate eg, into a mandatory consideration of criteria that reflect broad societal interest and consensus, like effectiveness, cost-effectiveness or severity of disease. Or, in countries where mandatory consideration of criteria is not pursued (or where they lack consensus on criteria that reflect broad societal interest) there is potential to learn from internationally endorsed societal values, that are the result of international and academic learning.

## How to Consider All Values in the Decision-Making Process?

When relevant values or key questions are identified throughout the process, they likely require further assessment. This may take the shape of (systematically) reviewing the literature, or creating an evidence-base for values that are quantifiable – eg, if health maximization is valued, then the technology’s performance can be assessed quantitatively by means of CEA, or for severity of disease by calculating the associated proportional shortfall.^20^ Values that are more difficult to quantify may be subjected to qualitative analysis, ie, ethical analysis or (expert) stakeholder opinions, to the extent possible. In the decision-making process, these pieces of quantitative and qualitative evidence – in conjunction with other values – should be deliberated on. The focus should be on finding out how these different pieces of information relate to each other and includes the making of value judgments about the available information. Again, this implies that appraisal and assessment go hand in hand throughout the process – constantly building and progressing towards a coherent and legitimate decision – and are not organized as separate phases. In a democratic society, political accountable decision-maker(s) hold the final authorizing decision-making power and therefore, stay accountable, not only for taking the final (or provisional) decision, but also for the deliberative procedures and actions that have been undertaken to come to this decision. However, this then warrants clarification on how responsible public decision-makers proceed from being one of the stakeholders – representing their own (public) interests – to taking the final decision in a fair way. How to best achieve this is yet unclear.

## How to Communicate the Argumentation – and Learning – That Led to the Final Decision?

It is important that all argumentation – on the use (or rejection) of criteria and their importance – is documented and made explicit in documents that serve to explain the decision to those who did not participate themselves. It is essential that not only the final result and its main rationales are spelled-out, but explanations why other identified concerns were not included should also be made explicit. Doing so in accountable ways will increase the likelihood that citizens who did not participate – and did not go through a learning process – can follow the complete reasoning underlying to the final decision. This allows for vicarious learning,^4^ and makes it more likely that they confer legitimacy to the process – and consider the decision as fair and legitimate – for well-justified reasons. Also, it may prevent that involved stakeholders shy away from making well-justified compromises that they would otherwise feel burdened to communicate in a convincing way to the ones they represent.

## Current Deliberative Approaches

A number of approaches for priority setting concentrate on evidence-informed deliberative processes, including the accountability for reasonableness (A4R),^2,21^ multi-criteria decision analysis (MCDA)^22^ and programme budgeting and marginal analysis (PBMA) frameworks.^23,24^ All consider deliberative processes important to develop and interpret the evidence-base for decision-making on the basis of stakeholder values and interests and to a certain extent foster learning among stakeholders. Interactive technology assessment (iTA)^12,13^ is specifically tailored to the concept of ‘HTA as learning’ and deliberative decision-making and is currently only in its experimental stage. Other approaches include citizen juries^25^ or round table conferences where stakeholders (eg, medical professionals, healthcare providers, health purchasers) are induced to reflect upon the technology at stake.^18^ We see large potential in the integration of such approaches to support evidence-informed deliberation and learning among stakeholders.

As an example, we have recently operationalized, implemented and evaluated a new process to guide priority setting in HIV/AIDS^26^ in West Java, Indonesia that integrates the A4R and MCDA frameworks.^27-29^ The overall aim of the process was to organise priority setting as an interactive learning process. A recent evaluation indicated that involved stakeholders were overall positive about the process, as it had improved the quality of decision-making – especially in terms of use of multiple criteria and concrete evidence, the active participation of stakeholders, and transparency of decision-making.^27,29^

Also, the Dutch National Health Care Institute is now introducing a scoping exercise in their decision-making process. During this scoping exercise stakeholders are consulted to help determine relevant outcome measures for the effectiveness of an intervention, which is a first step towards overcoming the assessment – appraisal split.^14,20^ Furthermore, a recent exploration of the potential role for MCDA in the Dutch decision-making process, showed that more deliberative forms of MCDA have potential to help structure deliberation among stakeholders.^15^

## Research Agenda

We see the following research agenda as key for progressing towards evidence-informed deliberative processes in priority setting:

- The use of a deliberative fair process is based on the assumption that stakeholders learn throughout the process and adjust their perspectives on the importance of criteria. Research – in the form of case studies – is needed that qualifies this assumption and evaluates learning by stakeholders and its impact on the final decision.
- Further research is needed on the translation of the practical lead ways as identified in this paper into organizational processes of HTA agencies. This relates to questions as: how can the concept of learning be best integrated into already existing deliberative approaches? How can processes be organized such that they can properly involve stakeholders?^30^ What kind of skills and expertise are needed throughout the process to make it a success, eg, interviewing skills to elicit stakeholder’ values? And how can this be organized within the time limits for decision-making? How can responsible decision-makers best guarantee that the final decision is taken fairly, considering they themselves are first actively participating in deliberation? How can decisions be best documented, reported and communicated to the general public, eg, with empathy, and in an understandable way? To answer these questions, it may be instrumental to review institutionalized deliberative processes in other sectors in society, eg, law.^6^
- Research has produced several lists of ‘relevant’ criteria that decision-makers can use when making a decision.^31-33^ Each list claims to capture a basic, generic, core set of criteria. Research is needed that identifies whether such a default list is useful to decision-makers as a starting point for deliberation, and if so, what should be on the list and in what form it can be provided to decision-makers. Eg, do stakeholders need in depth explanations of these criteria, or do they only require a short overview? Should the list of criteria be accompanied by an overview of arguments in favor or against trading-off specific criteria, to help stakeholders deliberate and form their judgements on the relative importance of criteria? Do the information needs differ per stakeholder group?
- Research is needed on how the use of evidence-informed deliberative processes relates to the currently dominant theoretical welfare framework on priority setting. Eg, does the use of such processes lead to welfare maximization in the same sense?

## The Bigger Picture: Health Technology Assessment as Social Learning

Presently, HTA is focused on providing a solution to the temporary task in front of us, namely, the priority setting exercise for which a decision has to be made. However, if efforts are made to organize HTA processes such that they truly enable learning among stakeholders and help shape understanding, then the impact of HTA will reach beyond the specific decision at hand – helping us to refine our societal understanding of what is valuable about health technologies and health. As such, it can actually stimulate social debate – which is valuable in itself. Learning from stakeholders (including citizens) is also instrumental in the sense that their expertise may help decision-makers to alter the limiting conditions and constraints in such ways that (provisional) approval becomes in the realm of possibilities.^6^ Then, HTA will be instrumental to discovering means to push out the boundaries of the possible, creating access to health technologies that are judged valuable but would otherwise have remained unavailable.

In conclusion, if we truly want to establish fair processes for priority setting that take into account all relevant social values, then we must re-organize the HTA process as an ongoing learning process – by soliciting stakeholders’ values and expertise right from the start, organize evidence collection on the basis of these values, and allow an organized interactive dialogue between stakeholders on the need for additional evidence and its meaning throughout the process. It is time to set the stage for HTA as learning.

## Ethical issues

Not applicable.

## Competing interests

Authors declare that they have no competing interests.

## Authors’ contributions

MPJ, RB: conception, design, and drafting of the manuscript; MPJ, RB, JKH, and BB: critical revision of the manuscript for important intellectual content; RB: supervision.

## Authors’ affiliations

^1^Radboud Institute for Health Sciences, Radboud University Medical Center, Nijmegen, The Netherlands. ^2^Institute for Management Research, Radboud University, Nijmegen, The Netherlands. ^3^Institute of Health Policy and Management, Erasmus University Rotterdam, Rotterdam, The Netherlands.
